# Traits of patients seen via telemedicine versus in person for new-patient visits in a fertility practice

**DOI:** 10.1016/j.xfre.2021.04.001

**Published:** 2021-04-15

**Authors:** Vinita M. Alexander, Allison P. Schelble, Kenan R. Omurtag

**Affiliations:** Department of Obstetrics and Gynecology, Division of Reproductive Endocrinology and Infertility, Washington University, St. Louis, Missouri

**Keywords:** Telemedicine, fertility, new-patient visit

## Abstract

**Objective:**

To assess the differences in demographics, the likelihood of receiving treatment, and the clinical outcomes between new patients seen via telemedicine and those seen in person in an academic fertility practice.

**Design:**

Retrospective cohort study.

**Setting:**

University-based fertility clinic.

**Patient(s):**

All new patients seen via telemedicine between June 1, 2017, and February 29, 2020, were compared with an equal number of all new patients seen in person between May 1, 2019, and June 30, 2019.

**Intervention(s):**

None.

**Main Outcome Measure(s):**

The primary outcome was receiving treatment after a new-patient visit. Binary logistic regression analyses were performed to estimate the odds ratio for not receiving treatment according to distance to the clinic and duration of infertility. The secondary outcomes included treatment recommendation, time to treatment initiation, and time to positive pregnancy test (if achieved). In addition we assessed patient demographics and visit traits per patient encounter.

**Result(s):**

The telemedicine and in-person groups each contained 70 patients. The following were similar between the groups: age, body mass index, Area Deprivation Index, diagnosis made at the new-patient visit, and the number of clinic contacts before starting treatment. Compared with patients who had in-person new-patient visits, those who had telemedicine new-patient visits lived farther from the clinic (mean, 223.6 vs. 69.28 miles) and had a longer duration of infertility (mean, 41.9 vs. 19.49 months). No differences were noted between the groups in the following outcomes: percent that received treatment, time to treatment initiation, or time to pregnancy. Telemedicine new-patient visits were shorter than in-person new-patient visits (mean, 56.3 ± 9.1 vs. 59.3 ± 4.6 minutes) and less likely to contain documentation of height or weight.

**Conclusion(s):**

Telemedicine appears to be of particular interest to patients who live farther from clinics and have longer durations of infertility, and it could reduce visit times. New patients seen in person and those seen via telemedicine are equally likely to pursue treatment. Telemedicine consultation for new-patient visits is feasible in an academic fertility practice and may be especially useful during a pandemic and in non-pandemic times in areas with limited access to fertility specialists.

**Discuss:** You can discuss this article with its authors and other readers at **https://www.fertstertdialog.com/posts/xfre-d-20-00242**

Telemedicine, in which electronic communication methods such as videoconferencing are used to deliver health care services, can increase access to health care, especially in rural areas (1). Additionally, telemedicine may be cost effective, and patients report high satisfaction with this type of care (1, 2). Specifically in fertility clinics, one study in Spain found that implementing telemedicine for women presenting for fertility evaluation yielded a shorter time to testing and treatment (3).

Despite these benefits, several fertility clinics have been slow to adopt telemedicine. However, that all changed with the COVID-19 pandemic. Although statistics have not been published for fertility providers, the urgent care clinic at NYU Langone Health reported a 683% increase in the use of telemedicine between March and mid-April 2020 (4). Likewise, in our university-affiliated fertility clinic, approximately 90% of our new-patient visits are occurring via telemedicine during the pandemic. Given this high use of telemedicine, it is important to determine whether telemedicine is as beneficial as in-person care.

One important concern among reproductive endocrinology and infertility (REI) providers has been that patients seen by telemedicine in their new-patient visit may not pursue treatment. This is founded on the fact that subfertile patients have high rates of dropping out of fertility care (5). Additionally, a retrospective cohort study of 384 couples reported that the physical or psychological burden of treatment was the most frequent cause of in vitro fertilization (IVF) dropout (6). Clinicians know that clear communication is important to patients discussing sensitive topics such as fertility (7), and telemedicine could adversely affect communication between patients and providers. Additionally, some REI physicians have suggested that the lack of physical face-to-face interaction in telemedicine could handicap the clinician in making an accurate diagnosis and lead to greater patient loss to follow-up or dropout (8).

Prior studies have shown that lower socioeconomic status (SES) may be associated with less access to fertility care (9) and lower pregnancy rates after IVF (10). The Area Deprivation Index (ADI) is a scale that may serve as a proxy measure for SES and takes into account geographically based social determinants of health (11). It is a validated measure quantifying neighborhood disadvantage and it is published online in the University of Wisconsin Neighborhood Atlas, which was derived from 2009–2013 US Census American Community Survey data (11). The ADI sorts neighborhood disadvantage percentile scores at the national level, with a higher ADI signifying increasing disadvantage. The ADI has previously been used in research evaluating the relationship between neighborhood disadvantage and 30-day readmission rates (13) as well as diabetes prevalence (14).

To our knowledge, no studies have investigated the utility of telemedicine in REI practices in the United States. We are in a good position to address this question because our clinic is located in Missouri, where those in rural areas have little access to fertility providers. This is because eight of the 10 IVF centers in Missouri are located within 30 miles of the state's two largest cities, St. Louis and Kansas City (16). To meet the demand for services outside of these cities, in 2017, Washington University Fertility and Reproductive Medicine Center began offering telemedicine consultations in conjunction with the regional hospital partner CoxHealth, which serves Southwest Missouri and Arkansas. Thus, we are able to examine the utility of telemedicine from the time before the COVID-19 pandemic. Here, our primary objective was to compare the rates of receiving treatment among patients whose new-patient visit was in person versus those among patients whose new-patient visit was via telemedicine. Our secondary objective was to evaluate the differences between demographic factors, time to treatment initiation, and clinical outcomes between those seen in person and those seen via telemedicine.

## Materials and methods

### Appointment Procedure at Our Practice During the Study Period

Between June 1, 2017, and February 29, 2020, if patients who called to schedule a new-patient visit lived in the region served by CoxHealth, the clinic staff told them about the option of scheduling an in-person or telemedicine visit. All patients seen in person coming from the CoxHealth region had previously been offered a telemedicine consultation. Patients who opted for telemedicine first signed an informed consent form that described the risks, benefits, and alternatives of a telemedicine visit. They then logged into a secure online portal (InTouch Health Telehealth software, Santa Barbara, CA) at a designated appointment time (sometimes at a local clinic site) and video-conferenced with the physician.

### Study Design and Data Collection

This study was approved by the St. Louis Institutional Review Board of Washington University School of Medicine. This was a retrospective study of data from Washington University Fertility and Reproductive Medicine Center electronic medical records (EMR). We analyzed data from all new-patient telemedicine encounters between June 1, 2017, and February 29, 2020. We also analyzed all new-patient in-person visits between May 1, 2019, and June 30, 2019. This interval was selected because it was within the time period used for telemedicine visits. Additionally, recent changes in software and scheduling templates made it easy to identify in-person visits in the EMR during this time frame. The EMR was reviewed for age, distance from residential address to clinic address (estimated using the shortest routed distance according to Google Maps [Alphabet Inc., Mountain View, CA]), diagnosis, date of service, new-patient visit type (in-person or telemedicine), and other details of patient’s history, treatment recommendations, and follow-up. The residential address was used to find the ADI, which uses a patient’s zip code to provide a percentile for socioeconomic disadvantage by incorporating key employment, housing quality, and poverty measures (11). The covariate “number of clinic contacts before starting treatment” included the number of messages or phone calls made by the patient before initiating treatment. Treatment recommendations were categorized into the following: ovulation induction, controlled ovarian hyperstimulation, IVF, surgery, oocyte cryopreservation, or other (including intrauterine insemination only or no treatment). Race and ethnicity data were not included in the analysis because they were not available for a large proportion of patients.

### Data Analysis

Data were analyzed using IBM SPSS v27.0 software (IBM Corp., Armonk, NY, USA). The χ^2^ analyses by group were performed for independent variables, and *t* tests were performed by group for continuous independent variables among all patients scheduled for a new-patient visit and among the patients who attended their new-patient visit. A P value of <.05 was considered statistically significant. Among the group who followed up, received treatment, and became pregnant, multivariate logistic regression was performed to examine the effects of patient traits and new-patient visit type on whether the patient became pregnant. Multivariate logistic regression analysis was also performed to examine the effects of patient traits and new-patient visit type on whether the patient received treatment. Variables with *P*<.05 were entered as covariates into this multivariable model. *G∗Power* (12) was used to conduct a post hoc power analysis.

## Results

### Patient Characteristics

Between June 1, 2017, and February 29, 2020, 70 unique patients scheduled telemedicine new-patient visits with the Washington University Fertility and Reproductive Medicine Center. Based on the clinic Society for Assisted Reproductive Technology data and rate of treatment pursuance, it is estimated that 130 patients from the region served by CoxHealth scheduled new-patient consultations during the same time period, and roughly 54% (70/130) elected to schedule telemedicine consultations. Between May 1, 2019, and June 30, 2019, 70 patients scheduled in-person new-patient visits. Table 1 shows that the following characteristics were similar between patients scheduled for in-person and telemedicine new-patient visits: age, body mass index (BMI), national ADI by zip code, rate of canceling the new-patient visit, diagnoses, and the number of clinic contacts made before starting treatment. Patients seen via telemedicine had significantly longer mean durations of infertility, lived farther from the clinic, had shorter new-patient visits, and were less likely to have their vital signs (including height and weight) recorded during the visit than those seen in person.

**Table 1:** Characteristics of patients scheduled for a new-patient visit at a fertility clinic.

Characteristic	In person (n = 70)	Telemedicine (n = 70)	*P value*
Age, y	33.5 ± 5.0	33.2 ± 5.2	.72
Body mass index, kg/m^2^	29.6 ± 8.5	29.9 ± 7.1	.40
National Area Deprivation Index, percentile	50.1 ± 23.3	62.8 ± 19.9	.24
Duration of infertility, mo	19.5 ± 23.9	41.9 ± 60.5	.006
Distance to the clinic, miles	69.3 ± 227	223.6 ± 26.3	.007
New-patient visit canceled			.38
Yes	9 (12.9)	14 (20)	
No	61 (87.1)	56 (80)	
Diagnosis in new-patient visits			.76
Ovulation disorder	14 (23.0)	11 (19.6)	
Uterine or tubal factor	6 (9.8)	7 (12.5)	
Diminished ovarian reserve	4 (6.6)	4 (7.1)	
Male factor	8 (13.1)	13 (23.2)	
Unexplained	10 (16.4)	10 (17.9)	
Other (e.g., endometriosis and elective)	19 (31.1)	11 (19.6)	
Duration of new-patient visit, min	59.3 ± 4.6	56.3 ± 9.1	<.001
Vitals listed in note in new-patient visits			<.001
Yes	57 (93.4)	0 (0)	
No	4 (6.6)	56 (100)	

*Note:* Values are presented as mean ± standard deviation or number (percent).

### Follow-Up After New-Patient Visits

No differences were noted between the telemedicine and in-person groups in the following respects: treatment recommended, percent that received treatment, and time to treatment initiation. Nineteen women in the in-person group and 11 women in the telemedicine group became pregnant. Of those who became pregnant, no statistically significant difference was noted between groups in the time to pregnancy (Table 2). After adjusting for distance to clinic, no statistically significant difference was noted between the groups in pregnancy rate. When comparing the patients who achieved pregnancy with those who did not, no other significant differences in traits were found between the groups (Table 3). Post hoc power calculations (α level of .05, power of at least .80) indicated that our study was adequately powered to demonstrate medium- and large-sized effects of telemedicine (.5 and .8, respectively, according to Cohen’s *d* effect size conventions) (17).

**Table 2:** Treatment recommendations and clinical outcomes of patients after a new-patient visit.

Outcomes	In person (n = 61)	Telemedicine (n = 56)	*P value*
Treatment recommended			.47
Ovulation induction	23 (37.7)	27 (48.2)	
Controlled ovarian hyperstimulation	2 (3.3)	3 (5.4)	
In vitro fertilization	22 (36.1)	17 (30.3)	
Surgery	3 (4.9)	4 (7.1)	
Oocyte cryopreservation	9 (14.8)	2 (3.6)	
Other (e.g., intrauterine insemination only, none)	2 (3.3)	3 (5.4)	
Received treatment?			.07, .80^a^
Yes	35 (57.3)	41 (73.2)	
No	26 (42.6)	15 (26.8)	
No. of clinic contacts before starting treatment ^b^	2.7 ± 1.8	1.5 ± 1.1	.15
Time to treatment initiation, d^b^	74.82 ± 68.0	77.5 ± 104.8	.32, .77^a^
Became pregnant^b^	19 (54)	11 (27)	<.01
After adjusting for distance from the clinic			.12
Time to pregnancy, d^c^	176.1 ± 86.1	226.5 ± 210.4^a^	.37

*Note:* Values are presented as mean ± standard deviation or number (percent).

a After adjusting for distance from the clinic.

b Only includes those who received treatment.

c Only includes those who became pregnant.

**Table 3:** Patient trait and association with achievement of pregnancy (n = 30) or no pregnancy (n = 46, among patients who pursued treatment).

Patient trait	Pregnant (n = 30)	Nonpregnant (n = 46)	*P value*
Body mass index, kg/m^2^	30.1 ± 7.3	30.7 ± 7.5	.73
Age at initial appointment, y	33.4 ± 3.7	33.4 ± 5.4	.50
Distance to the clinic, miles	116.9 ± 102.8	170.7 ± 111.4	.003
National Area Deprivation Index	54.9 ± 18.9	57.1 ± 24.5	.49
Diagnosis			.39
Ovulation disorder	6 (20)	12 (26.1)	
Uterine or tubal factor	5 (16.7)	2 (4.3)	
Diminished ovarian reserve	0 (0)	4 (8.7)	
Male factor	3 (10)	10 (21.7)	
Unexplained	8 (26.7)	8 (17.4)	
Other (e.g., endometriosis and elective)	8 (26.7)	10 (21.7)	
Duration of infertility, mo	26.8 ± 32.1	24.0 ± 25.9	.26
Treatment type			.27
Ovulation induction	13 (43.3)	26 (56.5)	
Controlled ovarian hyperstimulation	2 (6.7)	2 (4.3)	
In vitro fertilization	8 (26.7)	14 (30.4)	
Surgery	3 (10)	1 (2.2)	
Oocyte cryopreservation	0 (0)	3 (6.5)	
Other (e.g., intrauterine insemination only, none)	4 (13.3)	0 (0)	
Time to treatment initiation, d	45.5 ± 47.4	55.02 ± 69.6	.08

*Note:* Values are presented as mean ± standard deviation or number (percent).

### Likelihood of Receiving Treatment

In our regression model, after controlling for distance to the fertility clinic and duration of infertility, no statistically significant difference in the likelihood of receiving treatment was noted between patients whose new-patient visits were via telemedicine and those whose visits were in person (Table 4).

**Table 4:** Multivariate analysis of the odds of not receiving treatment after a new-patient visit.

	β value	*P value*	Odds ratio (95% CI)	Adjusted odds ratio (95% CI)^a^
Distance to the clinic, miles	0.004	.02	1.004 (1.001–1.008)	
Duration of infertility, mo	0.008	.26	1.01 (0.99–1.02)	
New-patient visit type, telemedicine vs. in person	−0.41	.08	2.03 (0.93–4.42)	0.66 (0.10–4.52)

*Note:* CI, confidence interval.

a Adjusted for distance to the clinic and duration of infertility.

## Discussion

Taken together, our results suggest that telemedicine is an effective strategy for new-patient visits in a fertility clinic. Our results demonstrated that no significant differences were noted between the demographics of patients seen via telemedicine or in person with respect to age, ADI (a surrogate marker for SES disadvantage in a patient’s region of residence), or infertility diagnosis. Telemedicine may be especially valuable to those living far from a fertility clinic, because we found that patients whose new-patient visits were scheduled via telemedicine lived farther from the clinic than those seen in person. This is important given that Harris et al. (18) estimated that approximately 18.2 million women of reproductive age in the United States lived in an area without an IVF clinic. In addition our data indicate that telemedicine may especially appeal to those with a long duration of infertility. Such patients may deem their prognosis for pregnancy bleak and abandon their quest to seek medical care after considering the logistical burdens of travel for a face-to-face encounter with a fertility specialist.

One concern in telemedicine is whether patients will understand physicians’ directions regarding the next steps to be taken. However, we found that the number of times the patients contacted the clinic before starting treatment was similar between those whose new-patient visits were via telemedicine and those whose visits were in person. Thus, telemedicine seems to be an effective means of conveying information to patients.

Another important concern for REI clinics is whether or not patients will pursue treatment after a telemedicine visit. Reassuringly, we found that even after controlling for distance to the clinic and duration of infertility, patients seen via telemedicine and those seen in person were equally likely to receive treatment. After adjusting for distance from the clinic, the pregnancy rates also appeared similar between the groups. Thus, despite the sensitive nature of fertility topics, the lack of face-to-face interaction in telemedicine does not seem to reduce patient follow-up.

We noted that patients seen via telemedicine had shorter appointment times than those seen in person. Although this difference was, on average, only three minutes, a physician seeing 10 patients in a day could save up to 30 minutes. However, we also noted that patients seen via telemedicine were less likely to have their vitals, including height and weight, documented during their visit than those seen in person. Specifically, 93.4% of patients seen in person had their current height and weight documented at the time of the new-patient encounter, while no patients seen via telemedicine had this information recorded. It is important that physicians are aware of a patient’s risk profile, including BMI, when designing treatment. If the precise BMI is not known or is not clarified during the course of a visit, an obese patient may be inappropriately counseled that she is safe to pursue pregnancy or IVF procedures. When such information is known upfront, a physician may be more apt to encourage a patient to pursue weight loss efforts before pursuing conception, especially if the patient is young, or at least caution them about the maternal and fetal risks of obesity in pregnancy. Thus, physicians should make sure to ask patients about their height and weight during telemedicine visits. Additionally, if fingertip detectors on smartphones become more widely available, physicians will be able to collect other vital data such as heart rate and blood pressure during a telemedicine visit. Multiple validation studies have demonstrated good agreement between self-reported and measured height, weight, and BMI in the general and obstetric populations (19, 20), and REI physicians should, thus, feel confident in collecting and counseling on this information via telemedicine.

Our study has several limitations. First, the telemedicine sample was limited to patients in the CoxHealth system, which covers a 25-county region of southwest Missouri and northwest Arkansas, who were offered and selected to be seen via telemedicine. Thus, our findings may not be generalizable to other geographic areas. Second, we did not have access to race or ethnicity data for all patients, so we cannot derive associations between race or ethnicity and the utility of telemedicine for new-patient fertility visits. Third, the in-person group was accrued over a shorter time period than the telemedicine group. Fourth, accrual for the telemedicine group began almost two years before accrual for the in-person group, so the follow-up windows were not identical. Fifth, although power detected a large effect, this sample was undersized to detect a possibly clinically significant small-sized effect of telemedicine on treatment rates. Finally, this is a retrospective study without data on the patient experience. In the future, it would be helpful to explore fertility patients’ satisfaction with telemedicine. The strengths of our study include the unique subject matter and access to a moderate volume of patients seen via telemedicine with a 3-year follow-up. Additionally, this is, to our knowledge, the first US study aiming to evaluate the traits of fertility patients selecting telemedicine for their new-patient fertility visit.

This study shows that telemedicine increases remote access to reproductive specialists with similar fertility outcomes as in-person care. However, the findings also serve as a reminder that the initial telehealth encounter may place more onus on the provider to obtain information like height, weight, and vital signs (rather than a medical assistant, who may document such information during an in-person visit). Historically, one barrier to the widespread usage of telemedicine was uncertainty about insurance coverage policies for the service. However, during the COVID-19 public health emergency, the Centers for Medicare and Medicaid Services took steps to enhance reimbursement telehealth services (15). Given the dramatic increase in telemedicine usage in the post-COVID-19 landscape, REIs should be encouraged by ongoing reimbursement rates for telehealth but should also prepare innovative strategies should reimbursement models change after the pandemic. However, it should also be kept in mind that it is incumbent on the REI specialist to inquire about history, height, and weight during the initial telehealth visit to uncover pertinent risk factors before making an initial treatment plan.

## Conclusion

Our study shows that telemedicine can facilitate access to fertility care for patients living far from a fertility clinic. Telemedicine also appears to be of particular interest to patients with long durations of infertility. Greater uptake of telemedicine in REI could reduce visit times. Importantly, patients seen in person or via telemedicine are equally likely to pursue treatment. However, telemedicine visits are less likely to collect information about vital signs, which could negatively affect treatment planning. We conclude that although telemedicine gained traction during the COVID-19 pandemic, it will continue to be a valuable and feasible means of conducting new-patient visits, especially for those living far from an REI clinic.
